# Differential regulation of the *hmsCDE* operon in *Yersinia pestis* and *Yersinia pseudotuberculosis* by the Rcs phosphorelay system

**DOI:** 10.1038/srep08412

**Published:** 2015-02-12

**Authors:** Xiao-Peng Guo, Gai-Xian Ren, Hui Zhu, Xu-Jian Mao, Yi-Cheng Sun

**Affiliations:** 1 MOH key laboratory of Systems Biology of Pathogens, Institute of Pathogen Biology, Chinese Academy of Medical Sciences and Peking Union Medical College, 9, Dongdan Santiao, Dongcheng District, Beijing, 100730, China

## Abstract

*Yersinia pestis*, the agent of plague, forms a biofilm in its flea vector to enhance transmission. *Y*. *pestis* biofilm development is positively regulated by *hmsT* and *hmsD*, encoding diguanylate cyclases (DGCs) involved in synthesis of the bacterial second messenger c-di-GMP. *rcsA*, encoding an auxiliary protein in Rcs phosphorelay, is nonfunctional in *Y*. *pestis*, while in *Yersinia pseudotuberculosis*, *rcsA* is functional and represses biofilms. Previously we showed that Rcs phosphorelay negatively regulates transcription of *hmsT* in *Y*. *pestis* and its ancestor *Yersinia pseudotuberculosis*. In this study, we show that Rcs positively regulates *hmsCDE* operon (encoding HmsD) in *Y*. *pestis*; while in the presence of functional *rcsA*, Rcs represses *hmsCDE* operon in *Y*. *pseudotuberculosis*. Loss of *rcsA*'s function in *Y*. *pestis* not only causes derepression of *hmsT* but also causes activation of *hmsD*, which may account for the increased biofilm formation in *Y*. *pestis*. In addition, differential regulation of the two DGCs, HmsT and HmsD by Rcs may help *Y*. *pestis* to adapt to different environment.

Y*ersinia pestis*, the causative agent of plague, is transmitted to mammals by infected flea bites. Transmission of *Y*. *pestis* is greatly enhanced after it forms a bacterial biofilm in the proventriculus of the flea^1^. *Y*. *pestis* biofilm is characterized by a dense aggregate of bacteria surrounded by a polysaccharide-rich extracellular matrix, which is synthesized and exported by the *Y*. *pestis hmsHFRS* genes^2^,^3^,^4^,^5^,^6^.

*Y*. *pestis* biofilms are positively regulated by cyclic-di-GMP (c-di-GMP), a second messenger existing in numerous gram negative bacteria^7^,^8^,^9^,^10^. C-di-GMP is synthesized by diguanylate cyclase (DGC) enzymes and is degraded by phosphodiesterase (PDE) enzymes^11^,^12^,^13^. The *Y*. *pestis* genome encodes two DGCs, HmsT and HmsD, related to biofilm formation^7^,^8^,^9^,^10^. The two DGCs of *Y*. *pestis* play differential roles in environment-dependent biofilm formation. HmsT plays a major role on *in vitro* biofilm formation, while HmsD plays a more prominent role in producing proventricular-blocking biofilm in the flea^10^.

The Rcs phosphorelay, a signal transduction system conserved in Enterobacteriacea^14^,^15^, was involved in regulation of biofilm formation, pathogenesis, motility, as well as the general stress response^16^,^17^,^18^,^19^,^20^,^21^. Rcs consists of the transmembrane sensor kinase RcsC, the response regulator RcsB and the transmembrane protein RcsD, which functions as the phosphorelay intermediate between RcsC and RcsB^14^,^15^. RcsB controls gene transcription initiation, acting in homodimers, or together with auxiliary proteins including RcsA, BglJ, GadE and others^22^,^23^,^24^,^25^,^26^. Interaction of RcsB with the auxiliary proteins extends its regulatory repertoire and represents a special mechanism of transcriptional control in bacteria^27^.

*Y*. *pestis* and its closely related ancestor *Yersinia pseudotuberculosis* biofilms are negatively regulated by the Rcs phosphorelay system *in vitro*^24^,^28^. *rcsA* is a nonfunctional pseudogene in *Y*. *pestis*, while in *Y*. *pseudotuberculosis*, *rcsA* is functional and represses biofilms^24^,^29^. We previously reported that Rcs repressed *hmsT* transcription in *Y*. *pestis*^24^. In this study, we identified another regulatory target of the Rcs phosphorelay in *Yersinia*. We show that in *Y*. *pestis*, RcsB activates *hmsCDE* transcription; while with the functional auxiliary protein RcsA in *Y*. *pseudotuberculosis*, RcsB represses *hmsD* transcription.

## Results

### Functional RcsA represses the *hmsCDE* operon

We previously reported that *Y*. *pestis rcsA* is mutated to a nonfunctional pseudogene and that introducing the functional *Y*. *pseudotuberculosis rcsA* into *Y*. *pestis* results in decreased biofilm formation^28^. Further study showed that Rcs phosphorelay repressed transcription of the diguanylate cyclase gene *hmsT*^24^. However, *Y*. *pestis* with functional *rcsA* forms almost no biofilm in the digestive tract of fleas, whereas the *Y*. *pestis hmsT* mutant forms intermediate levels of biofilm in the digestive tract of flea^24^, suggesting that *rcsA* may regulate other target genes to repress *Yersinia* spp. biofilms.

**Figure 1:**
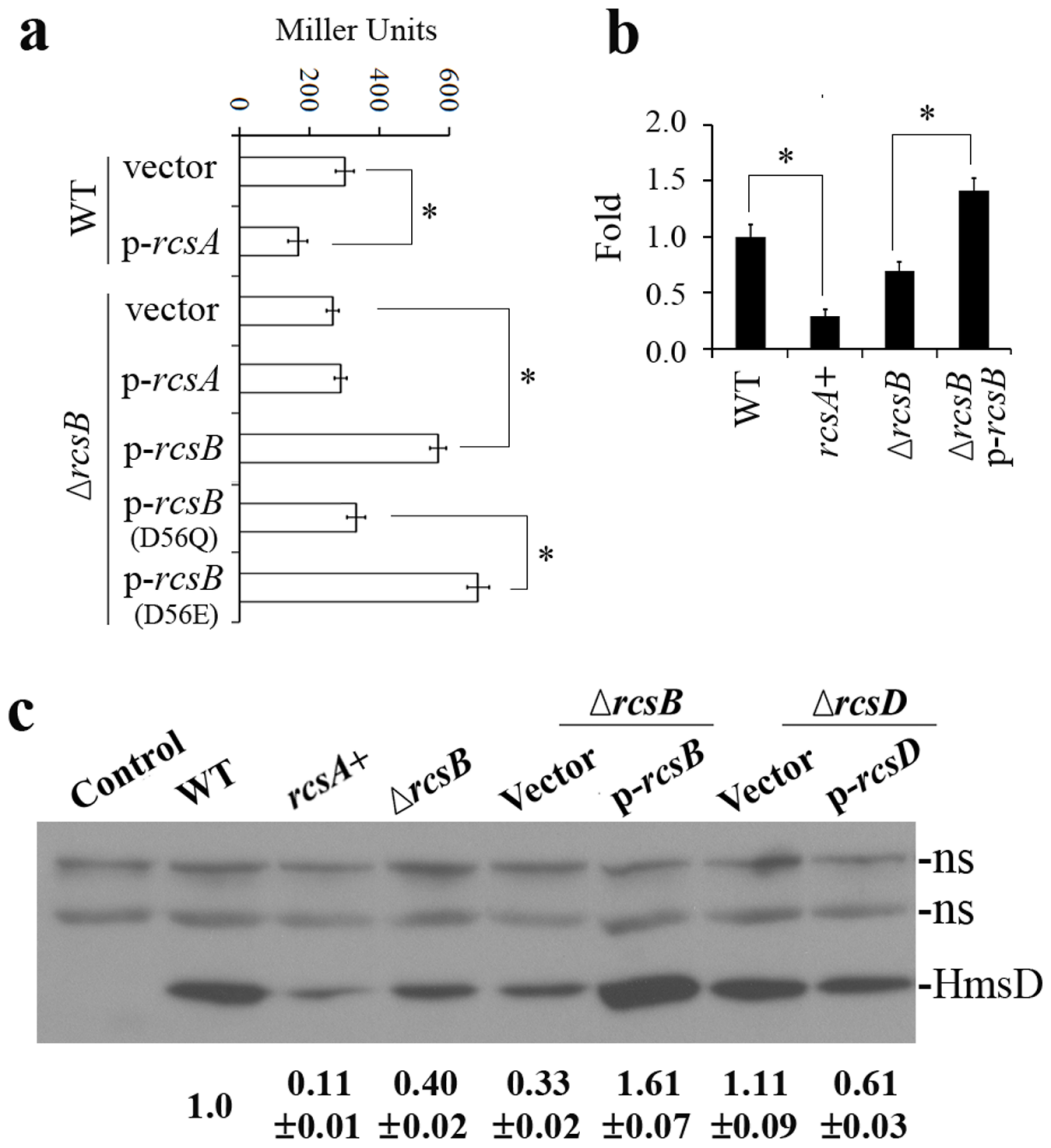
Regulation of *hmsCDE* by Rcs. (a) β-galactosidase activities of *hmsC*::*lacZ* reporter in *Y*. *pestis*. *Y*. *pestis* KIM6+ (WT) transformed with empty vector (vector) and functional RcsA (p-*rcsA*), RcsB deletion mutant transformed with empty vector (vector), functional RcsA (p-*rcsA*), wild-type RcsB (p-*rcsB*), inactive RcsB (p-*rcsB* (D56Q)) and active RcsB (p-*rcsB* (D56E)). (b) mRNA levels of *hmsD* regulated by Rcs in *Y*. *pestis*. *hmsD* mRNA levels were determined by qRT- PCR (Methods), and normalized to wild type. The mean and standard deviation of three independent experiments with three replicates are indicated. *P < 0.05. (c) Expression of HmsD regulated by Rcs in *Y*. *pestis*. Western blots of total protein-matched lysates prepared from stationary phase LB cultures and probed with anti-Myc antibody. Levels of HmsD were quantitated by densitometry using ImageJ from at least two independent experiments: numbers below blots indicate the ratio of HmsD from the indicated strain compared to that from wild-type *hmsD-*Myc_2_ strain (WT). ns, none specific band. Strain designations (Supplementary Table S1) are: Control, KIM6+ without Myc tag; WT, *hmsD-*Myc_2_; *rcsA+*, functional *rcsA hmsD-*Myc_2_; Δ*rcsB*, Δ*rcsB hmsD-*Myc_2_; Δ*rcsB* Vector, Δ*rcsB hmsD-*Myc_2_/pUC19; Δ*rcsB* p*-rcsB*, Δ*rcsB hmsD-*Myc_2_/pYC332; Δ*rcsD* Vector, Δ*rcsD*-N-terminal *hmsD-* Myc_2_/pET-32a; Δ*rcsD* p-*rcsD*, Δ*rcsD*-N-terminal *hmsD-* Myc_2_/pYC225. Full-length blots are presented in Supplementary Fig. S5 online.

Since *hmsD*, another DGC encoding gene in *Y*. *pestis*, plays a more important role in the biofilm formation *in vivo*^10^, we hypothesized that *hmsD* may be regulated by Rcs. To verify this hypothesis, we constructed transcriptional fusions using *E*. *coli lacZ* as the reporter. As *hmsD* is located in the *hmsCDE* operon, *hmsC*::*lacZ* was constructed in the pGD926 vector^30^. *hmsT*::*lacZ* and *lcrQ*::*lacZ* were constructed in the pGD926 vector as positive and negative controls. The resulting plasmids were transformed into *Y*. *pestis lacZ* mutants and assayed for β-galactosidase activity. As shown in Figure 1a and Supplementary Fig. S1, functional RcsA represses the transcription of *hmsC*::*lacZ* and *hmsT*::*lacZ* but not that of *lcrQ*::*lacZ*. To confirm this result of *lacZ* reporter assays, we tested the mRNA level of *hmsD* using the quantitative reverse transcription polymerase chain reaction (qRT-PCR). Transcription of *hmsD* is strongly decreased when functional RcsA is present (Figure 1b). We also examined HmsD expression by western blotting. Addition of 2× Myc (Myc_2_) to the C-terminus of HmsD does not affect its function^31^. The Myc_2_ C-terminal tag was introduced into the chromosome of *Y*. *pestis*. Consistent with the *lacZ* reporter assay and qRT-PCR result, HmsD is reduced in *Y*. *pestis* with functional *rcsA* (11% of the levels of wild type, Figure 1c, lane 3). Taken together, these results suggest functional *rcsA* repress the transcription of *hmsCDE* in *Y*. *pestis*.

### RcsB positively regulates the *hmsCDE* operon

We next used the *lacZ* reporters to determine the effects of RcsB. To our surprise, over-expression of RcsB produced a moderate increase while mutation of RcsB resulted in a slight decrease of the promoter activity of *hmsC* (Figure 1a). The positive control *hmsT*::*lacZ* was repressed while the negative control *lcrQ*::*lacZ* was not affected by over-expression of RcsB (Supplementary Fig. S1). qRT-PCR and western analysis confirmed that deletion of RcsB resulted in decreased expression of *hmsD*, while over-expression of RcsB activated the expression of *hmsD* (Figure 1b and c). Phosphorylation of aspartic acid 56 (D56) is usually required for RcsB's function. Replacement D56 with glutamic acid (E) can mimic the phosphorylated status of RcsB, while mutation of D56 with glutamine (Q) abolishes the phosphorylation of RcsB^32^,^33^. Over-expression of RcsB(D56E) but not RcsB(D56Q) results in induction of *hmsC* expression (Figure 1a), suggesting that phosphorylation of RcsB is necessary for the regulation of *hmsC*. Consistent with this and the previous finding that RcsD might dephosphorylate RcsB in *Y*. *pestis*^24^,^28^, deletion of *rcsD* resulted in slightly increased expression of HmsD (111% of the levels of wild type, Figure 1c), while over-expression of RcsD produced a moderate decrease in expression of HmsD (61% of the levels of wild type, Figure 1c). These results imply that RcsB positively regulates the *hmsCDE* operon.

**Figure 2:**
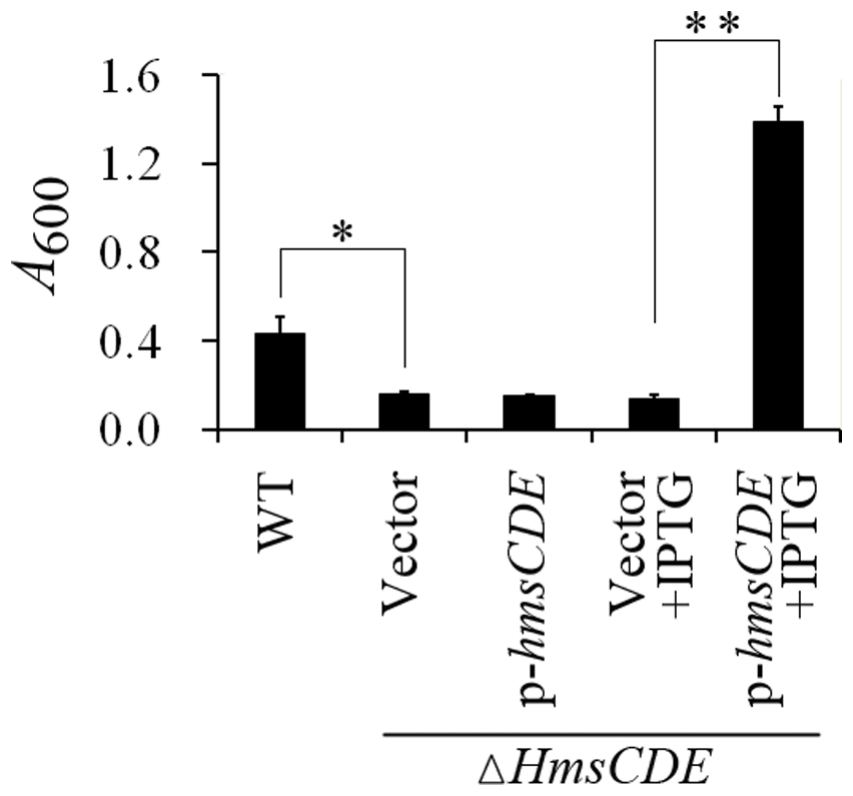
*hmsCDE* positively regulates biofilm formation in *Y*. *pestis*. *Y*. *pestis* biofilms produced in polystyrene culture dishes and quantified by crystal violet staining (Methods). The mean and standard deviation of three independent experiments with three replicates are indicated. *P < 0.05, **P < 0.01.

Since *hmsD* is located in the *hmsCDE* operon and since *hmsC* negatively regulates the function of *hmsD*^31^, we wanted to know the effect of expression of the *hmsCDE* operon on biofilm formation. To detect this, we cloned the *hmsCDE* genes into the pVTRA vector, where the *hmsCDE* genes are driven by an IPTG-inducible promoter. The resulting plasmid was transformed into the *Y*. *pestis hmsCDE* mutant and analyzed for biofilm formation. Consistent with previous findings that deletion of *hmsCDE* operon resulted in decreased biofilm formation, over-expression of *hmsCDE* following addition of IPTG resulted in increased biofilm formation (Figure 2), suggesting that increased transcription of *hmsCDE* activates biofilm formation. Taken together, these results suggest that Rcs might control biofilm formation in *Y*. *pestis* by upregulating the *hmsCDE* operon.

### An RcsAAB box in the *hmsC* promoter mediates the regulation of the *hmsCDE* operon by Rcs

Using two different 5′ RACE Kits (Methods), we determined the *hmsCDE* transcription start site to be 64-bp upstream of the initial ATG of *hmsC*. To verify this, we constructed a *lacZ* reporter with the mutated putative −10 box. The mutation of the −10 box resulted in nearly complete loss of activity of the *hmsC* promoter, indicating that the identified transcription start site is the sole or predominant one (Supplementary Fig. S2). To further rule out the possibility that *hmsD* has another promoter in the ORF of *hmsC*, we constructed a *lacZ* reporter that contained the *hmsC* ORF region but not its upstream sequence, and assayed for β-galactosidase activity. As shown in Supplementary Fig. S2, almost no promoter activity was detected in the analysis. Taken together, these results suggested that 64-bp upstream of ATG of *hmsC* is the sole or predominant transcription start site.

**Figure 3:**
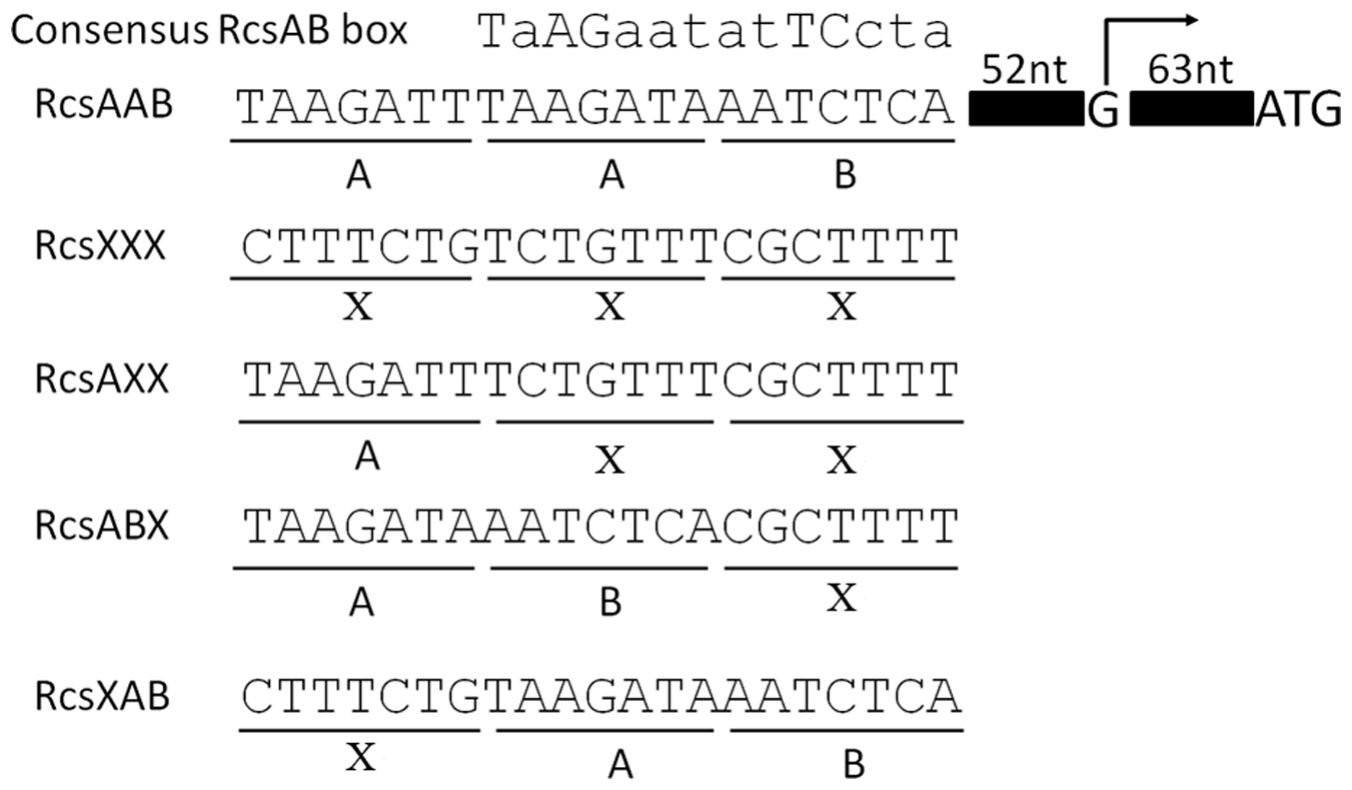
Organization of *hmsCDE* promoter-proximal region and the predicted or verified cis-acting elements. Top, the consensus RcsAB box TaAGaatatTCcta. The capital letters indicate conserved nucleotides. The right half is reported as the RcsB binding site, named B for short while the left part might be the RcsA binding site, named A for short. Middle, the organization of *hmsCDE* promoter-proximal region. Transcription start site G (+1) (under bent arrow) was mapped 64-bp upstream of the translation initiator codon ATG by 5′ RACE. By alignment with the consensus RcsAB box, the putative Rcs box of *hmsC* is located at 59-bp upstream of the transcription start site. A repeat of the left half of the conserved putative RcsA binding site is located immediately upstream of RcsAB box, thus it is designated as the RcsAAB box. Bottom, Rcs box mutations were introduced into the putative binding sites located at positions indicated. X indicates the binding site mutated.

An RcsAB box is located within 59-bp upstream of the *hmsC* transcription start site, which matches the consensus sequence at 9 of 14 nucleotides, including all five of the most conserved nucleotides(Figure 3). We noticed that the left half of the conserved RcsAB box is repeated immediately upstream of the RcsAB box. Since the right half of the RcsAB box is believed to be the RcsB binding site^23^,^26^,^34^, the left part might be the RcsA binding site. Thus, we designated this region as the RcsAAB box (Figure 3), which is found in all sequenced *Y*. *pestis* and *Y*. *pseudotuberculosis* (data not shown).

**Figure 4:**
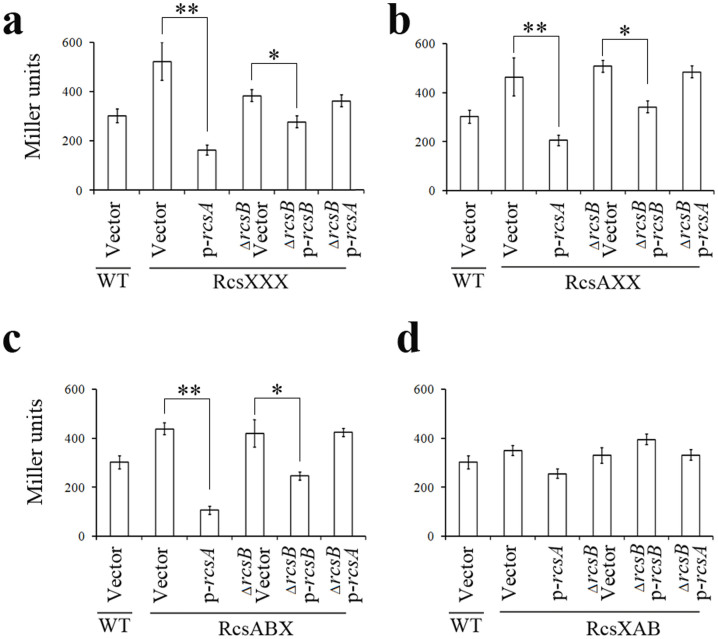
Role of the RcsAAB box on transcriptional regulation of *hmsCDE* by Rcs. β-galactosidase activities of *hmsC*::*lacZ* reporters with the RcsAAB box mutated to (a) RcsXXX, (b) RcsAXX, (c) RcsABX or (d) RcsXAB were analyzed. The mean and standard deviation of three independent experiments with three replicates are indicated. The mean and standard deviation of three independent experiments with three replicates are indicated. *P < 0.05, **P < 0.01.

To test the role of this region, we constructed a series of mutations of the RcsAAB box in the *hmsC*::*lacZ* fusion reporter (Figure 3) and analyzed the effects of Rcs on these mutated reporters in *Y*. *pestis*. Mutation of RcsAAB box to RcsXXX, RcsAXX or RcsABX but not RcsXAB, activated the *hmsC*::*lacZ* reporter. Functional *rcsA* strongly repressed the *hmsC* promoter with the RcsXXX, RcsAXX and RcsABX mutations in an *rcsB*-dependent manner (Figure 4a, b, c), but only slightly repressed the *hmsC* promoter with the RcsXAB mutation (Figure 4d), indicating that repression of *hmsCDE* by RcsA requires RcsB but is independent of the RcsAB binding site. To our surprise, RcsB also repressed *hmsC*::*lacZ* reporter when the RcsAAB box was mutated to RcsXXX, RcsAXX or RcsABX (Figure 4a, b, c), but slightly activated the *hmsC*::*lacZ* reporter in the RcsXAB mutant (Figure 4d). This result suggests that activation of *hmsCDE* by RcsB is dependent on the RcsB binding site in the RcsAAB box.

**Figure 5:**
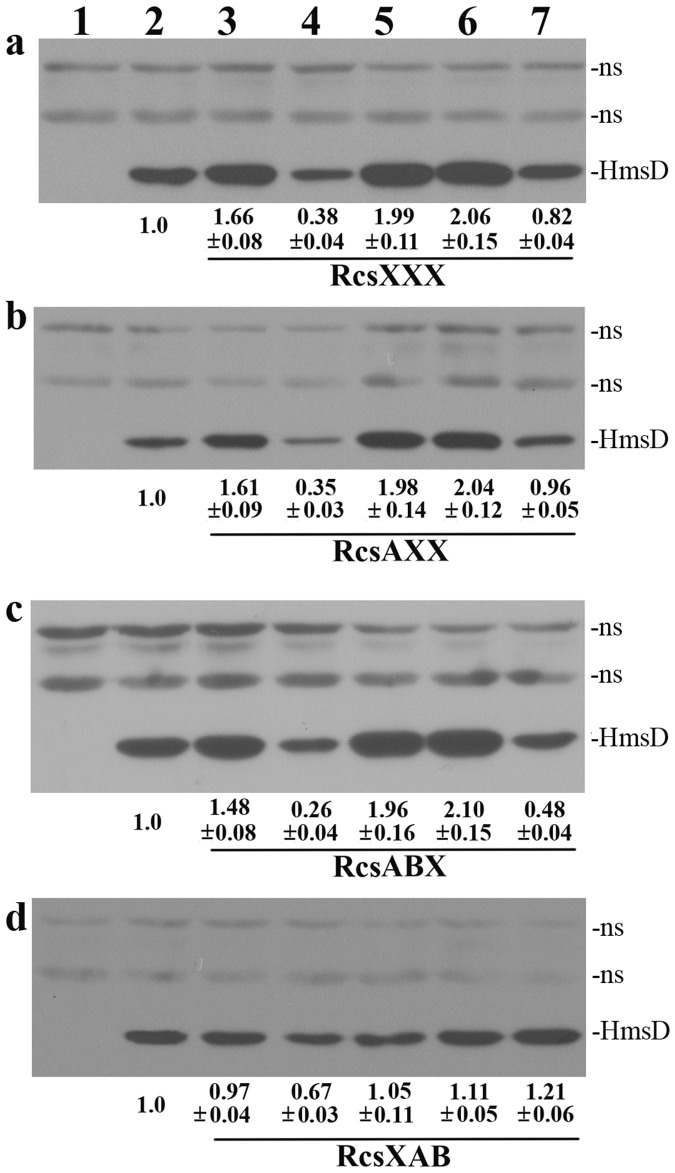
Role of the RcsAAB box on regulation of expression of HmsD by Rcs in *Y*. *pestis*. Western blots of total protein-matched lysates prepared from cells with mutation of (a) RcsXXX, (b) RcsAXX, (c) RcsABX or (d) RcsXAB were analyzed by anti-Myc antibody. Levels of HmsD were quantitated by using ImageJ from at least two independent experiments: numbers below blots indicate the ratio of HmsD from the indicated strain compared to that from wild-type *hmsD-*Myc_2_ strain (WT). Strain designations (Supplementary Table S1) are: 1, KIM6+; 2, *hmsD-*Myc_2_; 3, Rcs box mutation, *hmsD-*Myc_2_; 4, functional *rcsA*, Rcs box mutation, *hmsD-*Myc_2_; 5, Δ*rcsB*, Rcs box mutation, *hmsD-*Myc_2_; 6, Δ*rcsB*, Rcs box mutation, *hmsD-*Myc_2_/pUC19; 7, Δ*rcsB*, Rcs box mutation, *hmsD-*Myc_2_/pYC332. pYC332: p-*rcsB*. Full-length blots are presented in Supplementary Fig. S6–S9 online.

To confirm the results of the *lacZ* reporter assays, we further mutated the RcsAAB box on the chromosome and carried out western blotting analysis. Consistent with the *lacZ* analysis, HmsD expression was increased in *Y*. *pestis* with RcsXXX, RcsAXX and RcsABX (Figure 5a, b, c, lane 4) but not in *Y*. *pestis* with RcsXAB (Figure 5d, lane 4). Functional *rcsA* strongly repressed HmsD expression in *Y*. *pestis* with RcsAAB box mutation (Figure 5a, b, c, lane 4). RcsB repressed HmsD expression when the RcsAAB box was mutated to RcsXXX, RcsAXX or RcsABX (Figure 5a, b, c, lane 7), but slightly activated HmsD expression in *Y*. *pestis* with RcsXAB (Figure 5d, lane 7). The western blotting results further suggest that activation but not repression of HmsD expression by Rcs requires the RcsB binding site.

### *hmsD* promoter binding by RcsB

**Figure 6:**
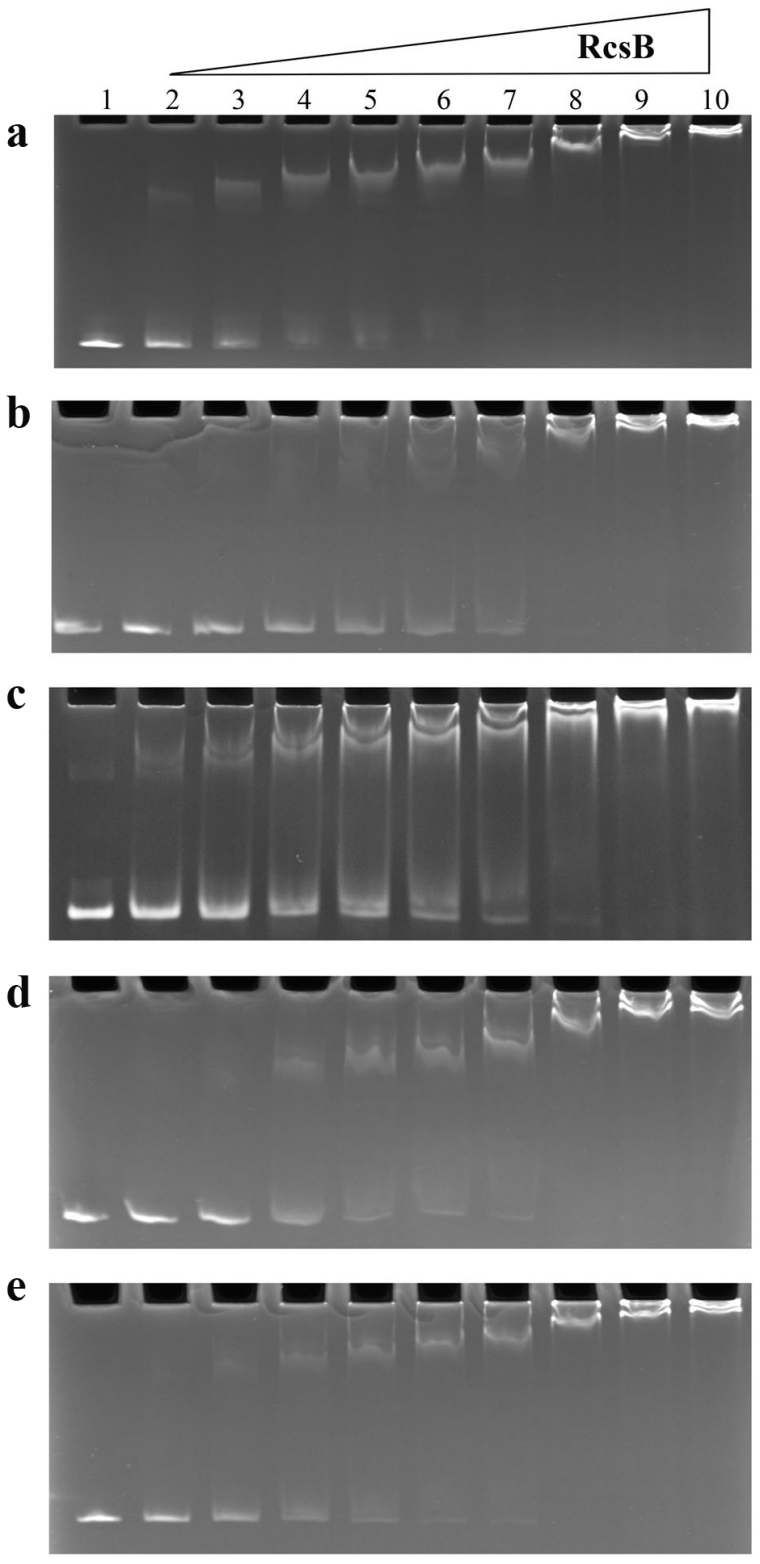
RcsB binds to the *hmsC* promoter. Electrophoretic mobility shift assays (EMSA) of *hmsC* promoter DNA incubated with increasing concentrations of RcsB. *hmsD* promoters with wild-type RcsAAB box (a) or with the mutated RcsAAB box RcsXXX (b), RcsAXX (c), RcsABX (d), or RcsXAB (e) were tested with identical protein combinations. Lane 1, *hmsD* probe alone; lanes 2–10, 100 ng *hmsCDE* probe with 400, 800, 1200, 1600, 2000, 2400, 4000, 6000 or 8000 ng of RcsB in the 16 μL reaction.

We directly assayed RcsB binding to the RcsAAB box using an electrophoretic mobility shift assay (EMSA). The DNA probe was a 112-bp promoter sequence comprising the RcsAAB box. The promoter DNA was electrophoresed alone or after incubation with purified RcsB (Methods). The promoter DNA of *hmsT* and *lcrQ* were analyzed by EMSA as positive and negative controls. Similar to *hmsT* promoter (Supplementary Fig. S3a), half of the *hmsD* probe was retarded by 800 ng of RcsB (Figure 6a, lane 3), and at 2000 ng the free probe was almost undetectable (Figure 6a, lane 6). In contrast, more than 2000 ng of RcsB was required to retard half of the *lcrQ* probe, and 4000 ng for essentially complete shifting (Supplementary Fig. S3b).To further examine the role of RcsAAB box, we also performed EMSA for RcsB with the mutated HmsD promoter. With the mutated RcsXXX or RcsAXX, more than 2000 ng of RcsB was required to retard half of the probe, and 4000 ng for essentially complete shifting (Figure 6b, c, lanes 6, 8), which is similar to that of the negative control *lcrQ* promoter (Supplementary Fig. S3b). However, with the mutated RcsXAB or RcsABX, about 1200 ng of RcsB was required to retard half of the probe, and 2400 ng for essentially complete shifting (Figure 6d, e, lane 5, 7).

### RcsB positively regulates but RcsAB negatively regulates *hmsD* in *Y*. *pesuodtuberculosis*

We next tested regulatory roles of RcsB and RcsA on *hmsD* in *Y*. *pesudotuberculosis* IP32953 using western blotting analysis. Consistent with the results in *Y*. *pestis*, RcsB mutation resulted in increased expression HmsD in *Y*. *pseudotuberculosis* (Supplementary Fig. S4a, lane 3). To our surprise, either mutation or over-expression of RcsB resulted in increased expression HmsD in *Y*. *pseudotuberculosis* (Supplementary Fig. S4a, lane 3, 7). To verify the role of the RcsB binding site, we also mutated the RcsAAB region to RcsAXX in *Y*. *pseudotuberculosis*. Consistent with the result in *Y*. *pestis*, repression of *hmsD* by Rcs was independent of the RcsB binding site (Supplementary Fig. S4b).

## Discussion

*Y*. *pestis* diverged from *Y*. *pseudotuberculosis* only 1,500-6,400 years ago^35^,^36^,^37^. We previously obtained evidence suggesting that, during the evolution of *Y*. *pestis*, mutation of *rcsA* was the product of natural selection and not genetic drift^28^. The mutation was required for *Y*. *pestis* to colonize its flea vector with a biofilm^28^,^29^. We also previously found that a target of Rcs regulation is *hmsT*^24^, encoding a DGC that regulates biofilms. Repressing *hmsT* apparently is not the only mechanism by which Rcs negatively regulates biofilms, because *Y*. *pestis hmsT* mutation and *Y*. *pestis rcsA-pstb* strains have similar biofilm phenotype *in vitro*, but different biofilm phenotypes *in vivo*^10^,^24^.

In this study, we showed that *hmsCDE*, encoding another DGC (*hmsD*) that regulates biofilms in *Yersinia*^28^,^31^, is also a target of Rcs regulation. Most importantly, we found a dual effect of the Rcs phosporelay on expression of *hmsD*: RcsB alone activates *hmsD* transcription, but RcsAB represses *hmsD* transcription. Several lines of evidence support this conclusion. First, *hmsD* transcription is reduced when functional RcsA is present (Figure 1a, b), and HmsD protein levels are also reduced (Figure 1c and Supplementary Fig. S4a, lane 2). Secondly, *hmsD* transcription is reduced when RcsB is mutated and stimulated when RcsB is over-expressed (Figure 1a), and HmsD protein levels are consistent with the transcriptional levels (Figure 1c). Thirdly, deletion of *hmsCDE* operon results in less biofilm formation but over-expression of *hmsCDE* activates biofilm formation (Figure 2). Finally, we showed that RcsB binds to the *hmsD* promoter in an RcsB binding site-dependent manner (Figure 6). Contrary to the finding that *rcsB* positively regulates *hmsCDE*, our previously result revealed that mutation of *rcsB* resulted in increased biofilm formation *in vitro*^24^,^28^. It can be explained by the fact that *hmsT* but not *hmsD* plays a dorminant role on *in vitro* biofilm formation^10^. Mutation of *rcsB* causes increased expression of *hmsT*^24^, which may account for the increased biofilm formation *in vitro*.

The Rcs phosphorelay signal transduction system is an important signaling pathway in the Enterobacteriaceae. It has been reported that RcsB functioned as an activator and also a repressor in the regulation of *gadA* in *E*. *coli*^23^. RcsB/GadE heterodimer binds to the GAD box, while RcsB alone binds to the RcsB box. Compared with the affinity of the RcsB/GadE for the GAD box, the affinity of RcsB for RcsB box is very low^23^. Thus, RcsB functions as an activator at lower concentration and as a repressor at high concentration in the regulation of *gadA*. Apparently it is not the same case for the regulation *hmsCDE*. An Rcs box is present at 59-bp upstream of the transcription start site of *hmsCDE*. This box is necessary for the activation but not the repression of *hmsCDE* transcription (Figure 4 and Figure 5). RcsB might bind to this box and then directly activate the transcription of *hmsCDE*.

The regulatory mechanism by which RcsAB represses *hmsCDE* transcription is still unknown. One hypothesis is that RcsAB acts indirectly by activating or repressing another regulator, which subsequently regulates *hmsCDE*. This hypothesis is supported by the facts that both RcsB and RcsAB negatively regulate *hmsCDE* expression when RcsAAB box is mutated to RcsXXX and RcsAXX (Figure 4 and Figure 5). In addition, there is still another question: why *Y*. *pestis* and *Y*. *pseudotuberculosis* have an RcsAAB box rather than an RcsAB box? One explanation is that the presence of left RcsA binding site affects the activation role of Rcs on *hmsCDE*. This hypothesis is supported by the result that Rcs regulation of *hmsCDE* is almost gone in the RcsXAB background (Figure 4d and Figure 5d).

**Figure 7:**
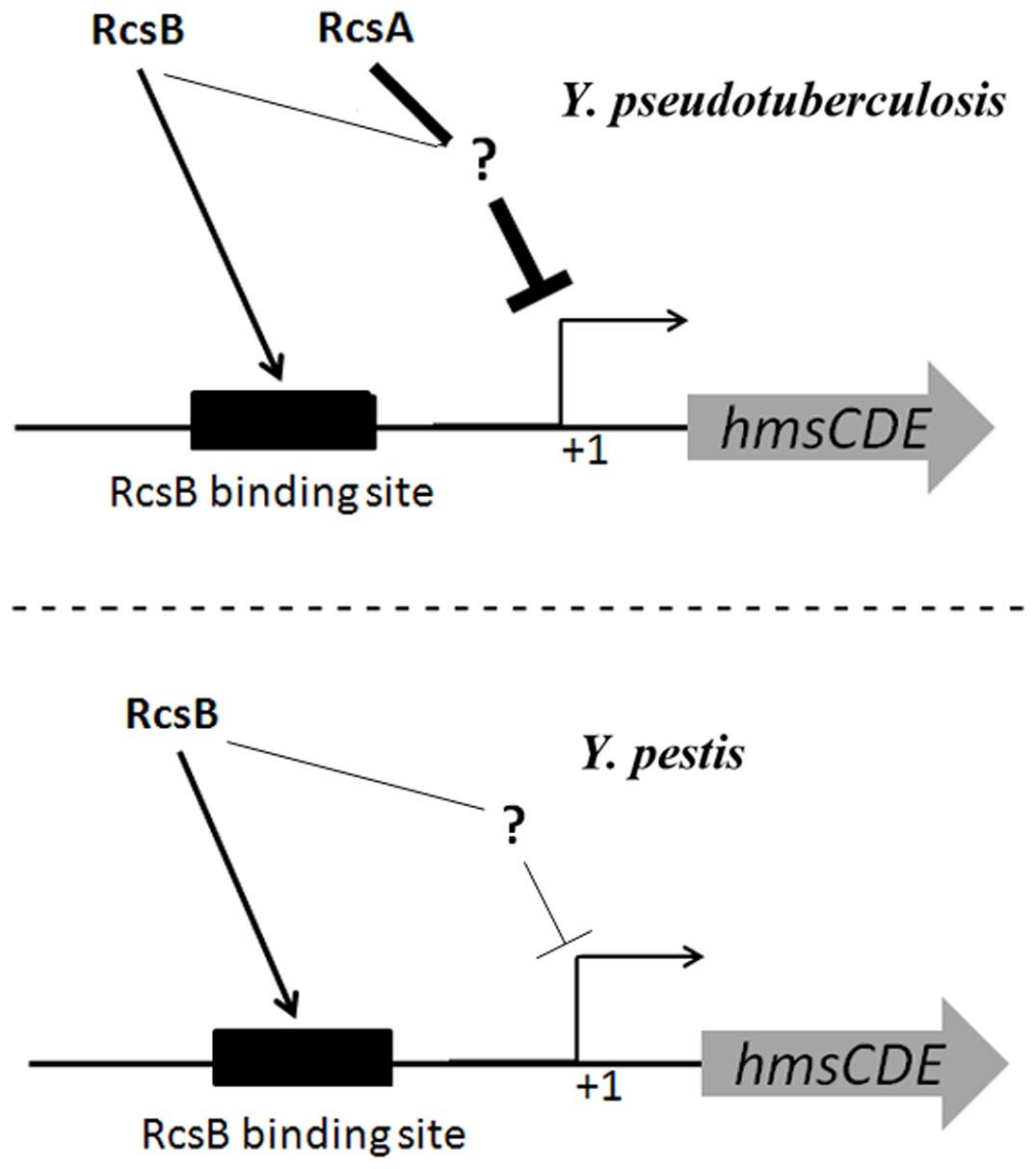
Regulation of *hmsCDE* transcription by Rcs in *Y*. *pseudotuberculosis* and *Y*. *pestis*. In *Y*. *pseudotuberculosis* (top), RcsB together with RcsA indirectly repress the transcription of *hmsCDE* independent of the RcsAB binding site, while RcsB activates transcription of *hmsCDE* dependent on the RcsB binding site. As a consequence, Rcs negatively regulates the *hmsCDE* operon in *Y*. *pseudotuberculosis*. In *Y*. *pestis* (bottom), as *rcsA* is mutated to nonfunctional pseudogene, RcsB alone directly positively but indirectly negatively regulates *hmsCDE* operon. As a consequence, Rcs positively regulates the *hmsCDE* operon in *Y*. *pestis*.

Our data support a model of multi-level control of *hmsCDE* by Rcs in *Yersinia* (Figure 7). In the presence of *rcsB* and functional *rcsA*, as is found in *Y*. *pseudotuberculosis*, RcsB together with RcsA repress the transcription of *hmsCDE* indirectly, which is independent of RcsAB binding site. At the same time, RcsB activates the transcription of *hmsCDE* in an RcsB binding site-dependent manner. As a whole, Rcs negatively regulates *hmsCDE* expression in *Y*. *pseudotuberculosis*. In the presence of *rcsB* alone, as found in *Y*. *pestis*, *rcsB* directly activates and indirectly represses *hmsCDE* expression. As a whole, Rcs negatively regulates *hmsCDE* expression in *Y*. *pestis*. In *Y*. *pseudotuberculosis*, *hmsT* and *hmsD* transcriptions are tightly repressed. This tight repression was relieved during *Y*. *pestis* evolution by the mutation of *rcsA* to a pseudogene^28^. There remains a residual repression of *hmsT* mediated by RcsB^24^, but expression of *hmsD* is activated by RcsB. We know that HmsT plays a dominant role on *in vitro* biofilm formation, while HmsD plays a major role on biofilm formation in the flea^7^,^10^. Thus RcsB negatively regulates *Y*. *pestis* biofilm formation *in vitro*, but might positively regulate biofilms *in vivo*. In summary, it appears that *Y*. *pestis* evolved to occupy the flea niche not only by derepressing *hmsT* but also by activating *hmsD*, thereby activating biofilm development.

The Rcs system might respond to different environmental signals, resulting in precise regulation of biofilm formation through control of *hmsT* and *hmsD*. In *Y*. *pseudotuberculosis*, RcsA and RcsB could respond to different environmental signals respectively and then precisely regulate *hmsCDE* transcription, which in turn control biofilm formation to adapt different environment. In *Y*. *pestis*, HmsT and HmsD are differentially regulated by Rcs, which may be partially responsible for the two DGCs played differential roles on environment-dependent biofilm formation^10^.

## Methods

### Bacterial strains and plasmids

The strains and plasmids used are shown in Supplementary Table S1. For construction of *hmsC*::*lacZ* and *hmsD*::*lacZ* reporter, the 353-bp upstream region together with the first 7 condons of the *hmsC* or the 624-bp upstream region together with the first 7 condons of the *hmsD* was amplified by PCR using KIM6+ chromosome DNA as template, respectively. The DNA fragments were digested with HindIII and BamHI, and cloned into pGD926^30^, resulting in plasmid pYC287 and pYC487. For construction of *hmsT*::*lacZ* and *lcrQ*::*lacZ* reporter, the 353-bp upstream region together with the first 7 condons of the *hmsT* or the 353-bp upstream region together with the first 7 condons of the *lcrQ* was amplified, digested, and cloned into pGD926, resulting in plasmid pYC593 and pYC597. RcsB(D56Q) and RcsB(D56E) were generated by overlapping PCR as described previously^33^.

The Rcs box mutation strains were made by two-step allelic replacement^38^, the mutation sequences are listed in Supplementary Table S2. Briefly, a 2-kb PCR product containing the promoter of *hmsCDE* was amplified from *Y*. *pestis* KIM6+ and cloned into pUC19. Using the resulting plasmid as template, the Rcs box was mutated using a mutagenic PCR primer, and the product was substituted into the *Y*. *pestis* chromosome by allelic replacement^38^. Oligonucleotide primers used are shown in Supplementary Table S2. All strains were verified by PCR, DNA sequencing or plasmid complementation, as appropriate.

### β-galactosidase assays

β-galactosidase activities were measured as previously described^24^. Overnight cultures of *Y. pestis* harboring *lacZ* reporters were diluted to OD_600_ 0.05 and grown in LB broth at room temperature to OD_600_ about 1.5. ONPG (o-nitrophenyl-β-D-galactopyranoside) was cleaved by lysates of cells at 37°C and expressed in Miller units^39^. Results from three independent experiments done in triplicate were analyzed.

### Quantitative real time PCR

qRT-PCR was carried out as previously described^10^. Briefly, cells were grown in LB broth overnight and diluted to OD_600_ 0.05 and grown in LB broth at room temperature to OD_600_ about 0.8. Total RNA was isolated from collected cells using the RNeasy Mini Kit (Qiagen). Residual DNA was removed by treatment with rDNase I (Ambion) and confirmed by PCR. cDNA was synthesized from the RNA and used for quantitative PCR on an ABI Prism 7900 Sequence Detection System (Taqman, Applied Biosystems). The quantity of mRNA was normalized relative to the quantity of the reference gene *crr* (y1485)^40^. The ratio of the normalized quantity of *hmsD* mRNA in different strains to the normalized quantity in the wild-type samples was calculated. Primers and probe sets used are listed in Supplementary Table S2. Results from three independent experiments done in triplicate were analyzed by one-way ANOVA with Bonferroni's test.

### Western blotting

Western blotting was carried out as previously described^24^. Samples were extracted from same amount of stationary phase cells cultured in 26°C, separated on 10% SDS-PAGE gels, transferred to PVDF membrane (Millipore), analyzed by immunoblot with antibodies to the Flag (Invitrogen) or Myc (Invitrogen), and detected with Immobilon Western HRP Substrate (Millipore). Results were quantitated by densitometry using NIH ImageJ.

### Transcription start site of *hmsCDE* operon

The transcription start site of *hmsCDE* gene was determined by using the 5′ RACE System for Rapid Amplification of cDNA Ends Kit (Invitrogen) and Smart®RACE cDNA Amplification Kit (Clontech) according to the manufacturer's instructions. Primer sequences are listed in Supplementary Table S2.

### Protein purification

RcsB was purified as previously described^24^. Briefly, *Y*. *pestis* strain containing pCBD209 was grown at 26°C to OD_600_ 0.6 and 0.2% arabinose was added to induce protein expression for 4 h. Cells were harvested by centrifugation and disrupted by sonication. The protein was then purified by using a Ni-nitrilotriacetic acid (NTA) His Bind Purification Kit (Novagen), as recommended by the manufacturer.

### Electrophoretic mobility shift assay (EMSA)

EMSA was performed as previously described. Briefly, a 112-bp PCR product containing the Rcs box or mutated Rcs box of the *hmsCDE* operon promoter region was amplified using the primers shown in Supplementary Table S2. Purified recombinant protein was added to DNA binding reaction mixtures containing 50 mmol Tris-HCl pH 7.5, 100 mmol NaCl, 10 mmol DTT, 500 μg/mL bovine serum albumin (BSA) and 100 ng PCR products. The binding assays were performed in a volume of 16 μL at room temperature for 30 min. After incubation samples were electrophoresed at 70 V for 1.5 hour in 6% DNA retardation gels. The gels were stained with ethidium bromide.

### *In vitro* biofilms

Microtiter plate biofilm assays were carried out as previously described^24^. Briefly, bacteria were cultured overnight in LB broth supplemented with 4 mmol CaCl_2_ and 4 mmol MgCl_2_ overnight and diluted to 96-well polystyrene plates, shaking for 24 hour at 26°C. The wells were washed and the adherent biofilm was stained with crystal violet, solubilized with 80% ethanol-20% acetone and measured by *A*_600_. Results from three independent experiments with five replicates per experiment were analyzed by one-way ANOVA with Dunnet's posttest to compare the wild type to the other strains.

## Author Contributions

Y.S. and X.G. wrote the main manuscript text. X.G. and G.R. prepared figures 1–6. H.Z. and X.M. prepared for some strains constructions. All authors reviewed the manuscript.

## Supplementary Material

Supplementary Information Supplementary information
