# Supplementary material for: Differential regulation of the hmsCDE operon in Yersinia pestis and Yersinia pseudotuberculosis by the Rcs phosphorelay system
Source: Sci Rep. 2015 Feb 12;5:8412. doi: 10.1038/srep08412 (PMC4325325; doi:10.1038/srep08412)
Supplement: Supplementary Information [file srep08412-s1.pdf]

Differential regulation of the *hmsCDE* operon in *Yersinia pestis* and *Yersinia pseudotuberculosis* by the Rcs phosphorelay system

Xiao-Peng Guo[1,2], Gai-Xian Ren[1,2], Hui Zhu[1], Xu-Jian Mao[1], Yi-Cheng Sun[1*]

[1]MOH key laboratory of Systems Biology of Pathogens, Institute of Pathogen Biology, Chinese Academy of Medical Sciences and Peking Union Medical College, 9, Dongdan Santiao, Dongcheng District, Beijing, 100730, China.

[2]These authors contribute equally to this work.

[*]Corresponding author: Yi-Cheng Sun.

email: sunyc@ipbcams.ac.cn; phone: 86 10 6783 7366

Supplementary Tables

Supplementary Table S1: Strains and plasmids used in this study

Strain or plasmid	Genotype and/ or description	Reference or source
Y. pestis		
KIM6+	wild type (pCD1-), nonfunctional *rcsA*	1
CDY326	Δ*rcsB*::*Km*	2
SY591	Δ*hmsCDE*	This study
SY1567	Δ*rcsD*-N-terminal, *hmsD*-Myc_2	This study
SY1568	Δ*rcsB*::*Km*, *hmsD*-Myc_2	This study
SY1570	*hmsD*-Myc_2	This study
SY1615	*hmsD*-Myc_2, RcsAXX[a]	This study
SY1726	functional *rcsA*, *hmsD*-Myc_2	This study
SY1783	functional *rcsA*, *hmsD*-Myc_2, RcsAXX	This study
SY1784	functional *rcsA*, *hmsD*-Myc_2, RcsABX[b]	This study
SY1785	*hmsD*-Myc_2, RcsXAB[c]	This study
SY1786	*hmsD*-Myc_2, RcsABX	This study
SY1788	Δ*rcsB*::*Km*, *hmsD*-Myc_2, RcsAXX	This study
SY1790	Δ*rcsB*::*Km*, *hmsD*-Myc_2, RcsABX	This study
SY1856	functional *rcsA*, *hmsD*-Myc_2, RcsXXX[d]	This study
SY1860	*hmsD*-Myc_2, RcsXXX	This study
SY1861	Δ*rcsB*::*Km*, *hmsD*-Myc_2, RcsXXX	This study
SY1910	Δ*rcsB*::*Km*, *hmsD*-Myc_2, RcsXAB	This study
SY1911	functional *rcsA*, *hmsD*-Myc_2, RcsXAB	This study
Y. pseudotu-berculosis		
IP32953	wild type; serogroup O1	3
CDY331	nonfunctional *rcsA*	2

SY1798	*hmsD*-Myc_2	This study
SY1799	nonfunctional *rcsA*, *hmsD*-Myc_2	This study
SY1937	Δ*rcsB*::*Km*, *hmsD*-Myc_2	This study
SY1939	nonfunctional *rcsA*, *hmsD*-Myc_2	This study
SY1966	*hmsD*-Myc_2, RcsAXX	This study
SY1967	nonfunctional *rcsA*, *hmsD*-Myc_2, RcsAXX	This study
SY1968	Δ*rcsB*::*Km*, *hmsD*-Myc_2, RcsAXX	This study
SY1969	Δ*rcsB*::*Km*, nonfunctional *rcsA*, *hmsD*-Myc_2, RcsAXX	This study
Plasmids		
pBAD/ Myc-His	expression vector, araBAD promoter, Amp^R	Invitrogen
pUC19	cloning vector for PCR products, Amp^R	4
pGD926	*lacZ* translational fusion vector, Tet^R	5
pVTRA	low level expression plasmid, IPTG induced, Cat^R	6
pCBD179	functional *rcsA* in pUC18	2
pCBD209	*rcsB* in pBAD/ Myc-His	7
pYC287	*hmsC* 5' UTR::*lacZ* fusion in pGD926	This study
pYC300	*hmsC* 5' UTR (RcsAXX)::*lacZ* fusion in pGD926	This study
pYC301	*hmsC* 5' UTR (-10 box*[e])::*lacZ* fusion in pGD926	This study
pYC332	*rcsB* in pUC19	This study
pYC484	*hmsC* 5' UTR (RcsXXX)::*lacZ* fusion in pGD926	This study
pYC485	*hmsC* 5' UTR (RcsXAB)::*lacZ* fusion in pGD926	This study
pYC486	*hmsC* 5' UTR (RcsABX)::*lacZ* fusion in pGD926	This study
pYC487	*hmsC*::*lacZ* fusion in pGD926	This study
pYC513	*hmsCDE* in pVTRA	This study
PYC575	*rcsB*(D56Q) in pUC19	This study
PYC576	*rcsB*(D56E) in pUC19	This study
PYC593	*hmsT* 5' UTR::*lacZ* fusion in pGD926	This study
PYC597	*lcrQ* 5' UTR::*lacZ* fusion in pGD926	This study

[a]RcsAXX: RcsAAB mutation showed as Figure 3: TAAGATT**TCTGTTTCGCTTTT** (the mutated base pairs are bold, the same below).

[b]RcsABX: RcsAAB mutation showed as Figure 3: TAAGATAAATCTCA**CGCTTTT**

[c]RcsXAB: RcsAAB mutation showed as Figure 3: **CTTTCTG**TAAGATAAATCTCA

[d]RcsXXX: RcsAAB mutation showed as Figure 3: **CTTTCTGTCTGTTTCGCTTTT**

[e]-10 box*: -10 box mutation of *hmsC* promoter.

Supplementary Table S2: Oligonucleotides used in this study

Construction of *rcsB* in pUC19 (pYC332)
cgggatccatttaagcagcgcggttat
ggaattctactacagtgaccaagtacggcg

Construction of *lacZ* deletion
atgacgtcacaggaaaaggtaccactccaggtgcaactgagtcttgtgtaggctggagctgcttcg
ttacaccttgtattgccaacagatttggtactgataggtttcacgcatatgaatatcctccttag

Construction of *hmsC* 5' UTR::*lacZ* fusion in pGD926 (pYC287)
cccaagcttgtccagcccagctccg
cgggatccgcgcaagtagcggtagtc

Construction of *hmsC*::*lacZ* fusion in pGD926 (pYC487)
cccaagcttatgactaccgctacttgcg
cgggatcctgatttttttcggcttcat

Construction of *hmsT* 5' UTR::*lacZ* fusion in pGD926 (pYC593)
caagcttcacggctgagcaacccgg
cgcggatccatattcaatttactctgca

Construction of *lcrQ* 5' UTR::*lacZ* fusion in pGD926 (pYC597)
ccaagcttgcgtttggatcaaggggg
cgcggatcctgaagagtattgattttcatcg

Determination of *hmsC* transcription start site
attctggcagcagcatcgctacgttcacgc
gcattgatattactggcggccaataatggggttggtac
agccaccaataatgaaagtaa
cgacgttattataagatttatatga

PCR amplification of *hmsC* promoter for EMSA
tacctgacaaaaacccggaaa
tgatgtaataatagtcatatcatcgt

PCR amplification of *hmsT* promoter for EMSA
tcatgatgacaggctgaaaca
tccgacatcacgacaaataaa

PCR amplification of *lcrQ* promoter for EMSA
cccaaaaataattttttattgtgattt
tataattgtctctacgatattctaagttatttatt

RcsB (D56Q) mutagenesis

aattactcaactctctatgccaggggataagtatggtgatggcatcac
agagttgagtaattagcacgttggcatcaagtttggacaaattgttaataagc

RcsB (D56E) mutagenesis

aattactgaactctctatgccaggggataagtatggtgatggcatcac
agagttcagtaattagcacgttggcatcaagtttggacaaattgttaataagc

RcsAXX box mutagenesis

tctgtttcgcttttaatttacgatgatatgactattattacatcatataaatcttataataacg
aaaagcgaaacagaaatcttatttatgttattgaaatagaaatataaaaaacccga

RcsXXX box mutagenesis

ctttctgtctgtttcgcttttaatttacgatgatatgactattattacatcatataaatcttataataacg
aaaagcgaaacagacagaaagtttatgttattgaaatagaaatataaaaaacccgactgtt

RcsXAB box mutagenesis

ctttctgtaagataaatctcaaatttacgatgatatgactattattacatcatataaatcttataataacg
tgagatttatcttacagaaagtttatgttattgaaatagaaatataaaaaacccgactgtt

RcsABX box mutagenesis

aatctcacgcttttaatttacgatgatatgactattattacatcatataaatcttataataacg
aaaagcgtgagattaatcttatttatgttattgaaatagaaatataaaaaacccga

-10 box mutagenesis

ttaggtacgacgttagatctagatttatatgatgtaataatagtcatatcatcgtaaattaagatctttctgtc
agatctaacgtcgtacctaacgccgattcaaccaccacattta

***hmsD* qRT-PCR**

acctgcatttgatcgcccgcg36-TAMSp
gccttacgggtttatgttgatcac
ggcctcggtggtataactgatg

***crr* qRT-PCR**

ctcctgttgacggcaccatcggt36-TAMSp
gccctctggcaataaaatgg
agcatggttggtctcgaaaatt

All sequences are in 5´-3´orientation.

Supplementary Figures

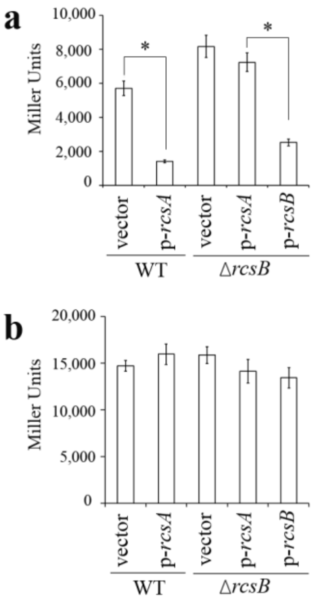

Supplementary Figure S1: β-galactosidase activities of *hmsT*::*lacZ* and *lcrQ*::*lacZ* reporter. β-galactosidase activities of *hmsT*::*lacZ* (a) and *lcrQ*::*lacZ* (b) reporter in *Y. pestis*. *Y. pestis* KIM6+ (WT) transformed with empty vector (vector) and functional RcsA (p-*rcsA*), RcsB deletion mutant transformed with empty vector (vector), wild-type RcsA (p-*rcsA*) and wild-type RcsB (p-*rcsB*). The mean and standard deviation of three independent experiments with three replicates are indicated. *P<0.01.

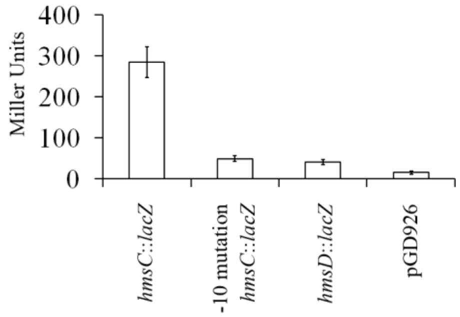

Supplementary Figure S2: β-galactosidase activities of *hmsC*::*lacZ* and *hmsD*::*lacZ* reporters. β-galactosidase activities of *hmsC*::*lacZ* and *hmsD*::*lacZ* reporters in *Y. pestis*. KIM6+ (WT) transformed with *hmsC*::*lacZ, hmsC*::*lacZ* with mutated putative -10 box, *hmsD*::*lacZ* and pGD926 plasmids. The mean and standard deviation of three independent experiments with three replicates are indicated. **P*<0.05, ***P*<0.01.

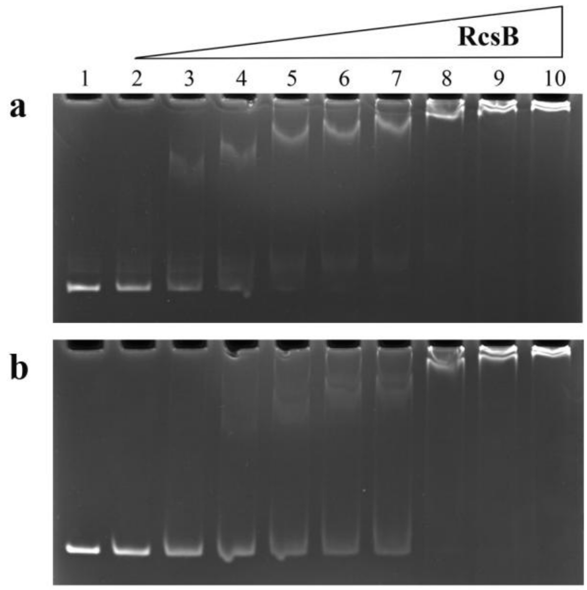

Supplementary Figure S3: EMSA results of RcsB with *hmsT* and *lcrQ* promoter.

Electrophoretic mobility shift assays (EMSA) of *hmsT* (a) and *lcrQ* (b) promoter DNA incubated with increasing concentrations of RcsB. Probes were tested with identical protein combinations. Lane 1, probe alone; lanes 2-10, 100 ng probe with 400, 800, 1200, 1600, 2000, 2400, 4000, 6000 or 8000 ng of RcsB in the 16 μL reaction.

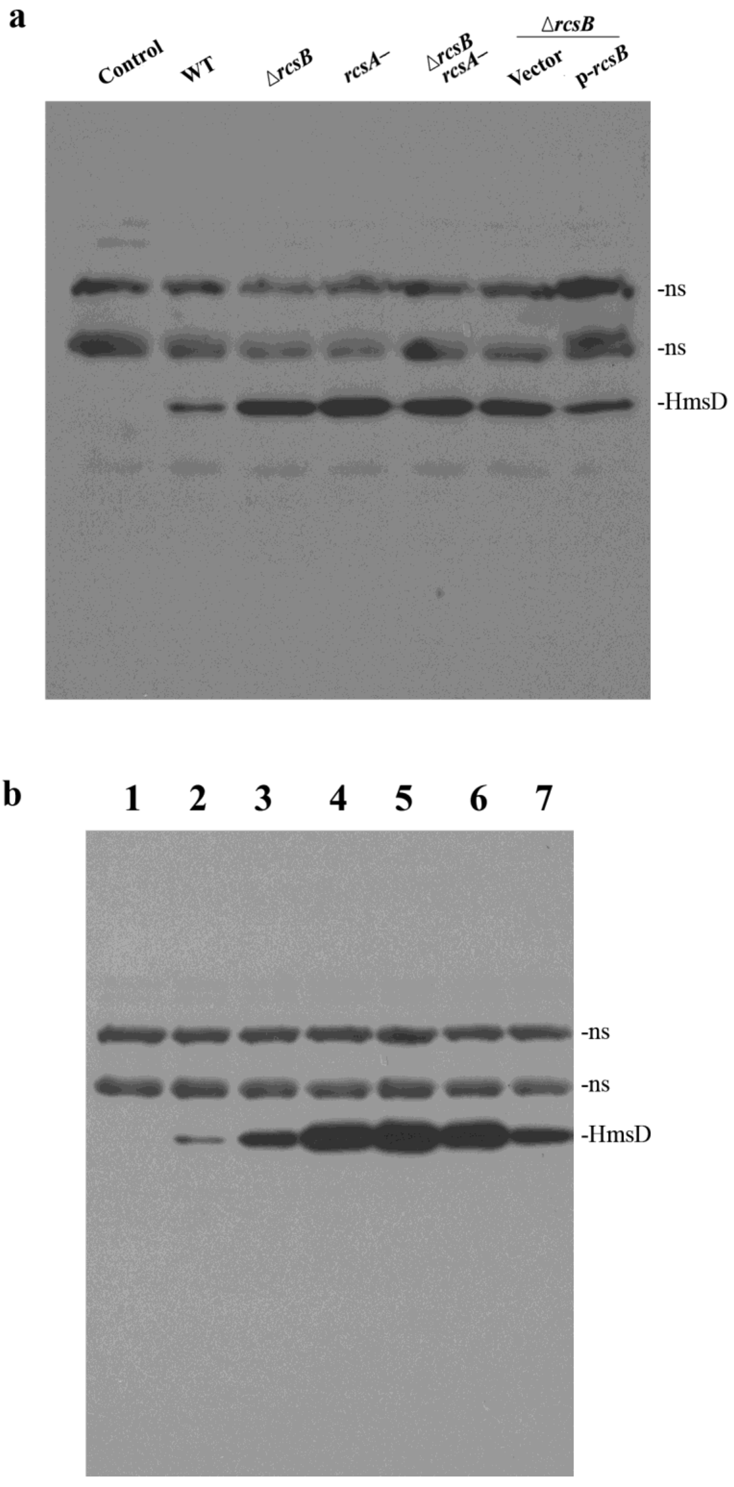

Supplementary Figure S4: Role of the RcsAAB box on regulation of expression of HmsD by Rcs in *Y. pseudotuberculosis*. Western blots of total protein-matched lysates prepared from cells with (a) RcsAAB box and (b) RcsAXX box were analyzed by anti-Myc antibody. Strain designations (Supplementary Table S1) are: (a) Control, IP32953; WT, *hmsD*-Myc$_2$; Δ*rcsB*,

Δ*rcsB hmsD*-Myc_2; *rcsA*-, nonfunctional *rcsA hmsD*-Myc_2; Δ*rcsB rcsA*-, nonfunctional *rcsA* Δ*rcsB hmsD*-Myc_2; Δ*rcsB* Vector, Δ*rcsB hmsD*-Myc_2/ pUC19; Δ*rcsB* p-*rcsB*, Δ*rcsB hmsD*-Myc_2/ pYC332.

(b) 1, IP32953; 2, *hmsD*-Myc_2; 3, RcsAXX mutation, *hmsD*-Myc_2; 4, nonfunctional *rcsA*, RcsAXX mutation, *hmsD*-Myc_2; 5, Δ*rcsB*, RcsAXX mutation, *hmsD*-Myc_2; 6, Δ*rcsB*, RcsAXX mutation, *hmsD*-Myc_2/ pUC19; 7, Δ*rcsB*, RcsAXX mutation, *hmsD*-Myc_2/ pYC332. pYC332: p-*rcsB*.

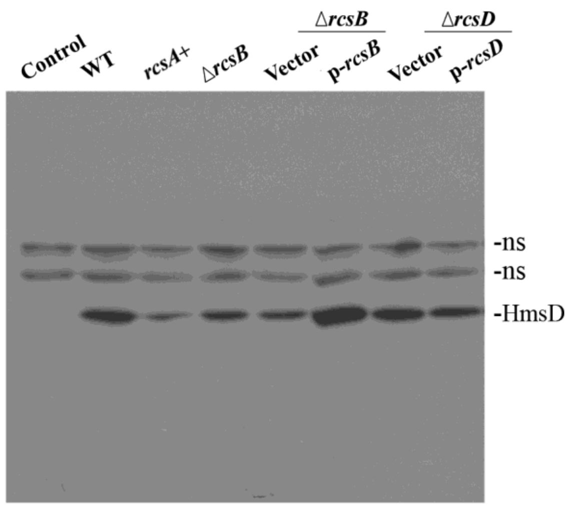

Supplementary Figure S5: Full-length blots for Figure 1c.

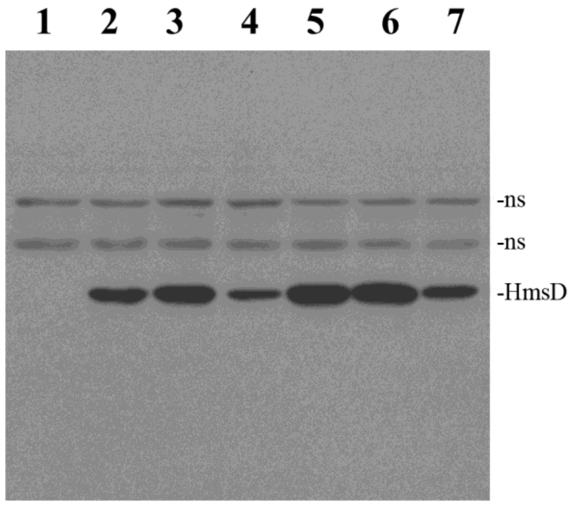

Supplementary Figure S6: Full-length blots for Figure 5a.

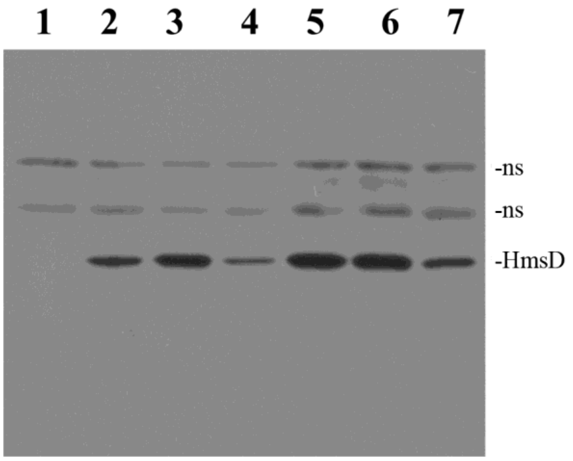

Supplementary Figure S7: Full-length blots for Figure 5b.

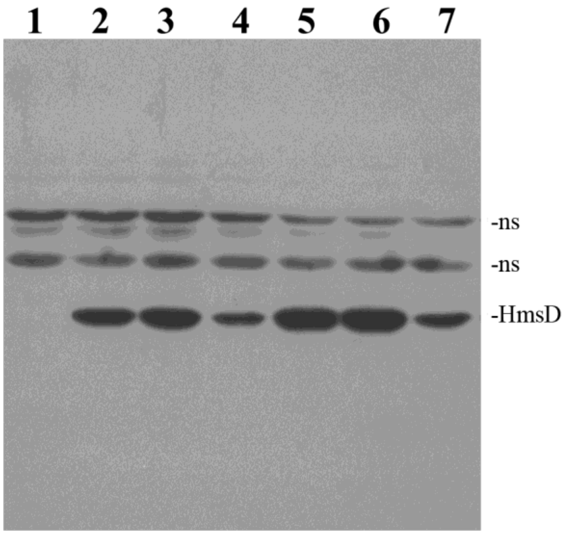

Supplementary Figure S8: Full-length blots for Figure 5c.

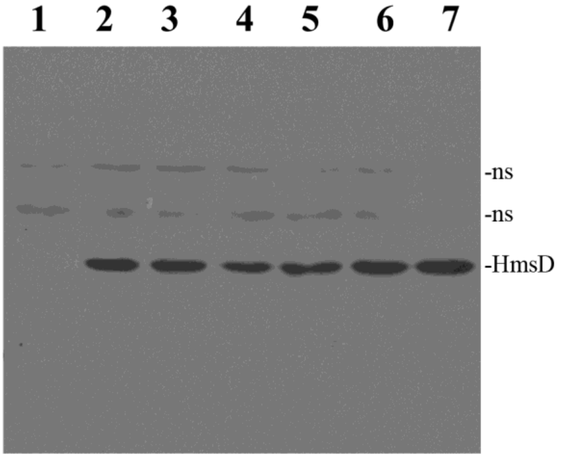

Supplementary Figure S9: Full-length blots for Figure 5d.

Supplementary References

1 Fetherston, J. D., Schuetze, P. & Perry, R. D. Loss of the pigmentation phenotype in *Yersinia pestis* is due to the spontaneous deletion of 102 kb of chromosomal DNA which is flanked by a repetitive element. *Mol. Microbiol.* **6**, 2693-2704 (1992).

2 Sun, Y. C., Hinnebusch, B. J. & Darby, C. Experimental evidence for negative selection in the evolution of a *Yersinia pestis* pseudogene. *Proc. Natl. Acad. Sci. U S A.* **105**, 8097-8101 (2008).

3 Chain, P. S. *et al*. Insights into the evolution of *Yersinia pestis* through whole-genome comparison with *Yersinia pseudotuberculosis*. *Proc. Natl. Acad. Sci. U S A.* **101**, 13826-13831 (2004).

4 Yanisch-Perron, C., Vieira, J. & Messing, J. Improved M13 phage cloning vectors and host strains: nucleotide sequences of the M13mp18 and pUC19 vectors. *Gene* **33**, 103-119 (1985).

5 Ditta, G. *et al*. Plasmids related to the broad host range vector, pRK290, useful for gene cloning and for monitoring gene expression. *Plasmid* **13**, 149-153 (1985).

6 Perez-Martin, J. & Lorenzo, V. D. VTR expression cassettes for engineering conditional phenotypes in *Pseudomonas*: activity of the Pu promoter of the TOL plasmid under limiting concentrations of the XylR activator protein. *Gene* **172**, 81-86 (1996).

7 Sun, Y. C., Guo, X. P., Hinnebusch, B. J. & Darby, C. The *Yersinia pestis* Rcs phosphorelay inhibits biofilm formation by repressing transcription of the diguanylate cyclase gene *hmsT*. *J. Bacteriol.* **194**, 2020-2026 (2012).
